# Membrane lipids in invadopodia and podosomes: Key structures for cancer invasion and metastasis

**DOI:** 10.18632/oncotarget.164

**Published:** 2010-09-07

**Authors:** Hideki Yamaguchi, Tsukasa Oikawa

**Affiliations:** ^1^Growth Factor Division, National Cancer Center Research Institute, Tokyo 104-0045, Japan; ^2^Precursory Research for Embryonic Science and Technology, Japan Science and Technology Agency, Saitama 332-0012, Japan; ^3^Collaborative Research Resources, Institute for Integral Medical Research, School of Medicine, Keio University, Tokyo 160-8582, Japan

**Keywords:** Invadopodia, podosome, cancer invasion, metastasis, lipid raft, phosphoinositide

## Abstract

Invadopodia are extracellular matrix (ECM)-degrading protrusions formed by invasive cancer cells. Podosomes are structures functionally similar to invadopodia that are found in oncogene-transformed fibroblasts and monocyte-derived cells, including macrophages and osteoclasts. These structures are thought to play important roles in the pericellular remodeling of ECM during cancer invasion and metastasis. Much effort has been directed toward identification of the molecular components and regulators of invadopodia/podosomes, which could be therapeutic targets in the treatment of malignant cancers. However, it remains largely unknown how these components are assembled into invadopodia/podosomes and how the assembly process is spatially and temporally regulated. This review will summarize recent progress on the molecular mechanisms of invadopodia/podosome formation, with strong emphasis on the roles of lipid rafts and phosphoinositides.

## INTRODUCTION

**Figure 1:**
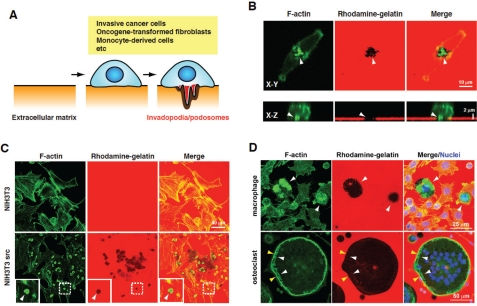
Invadopodia and podosomes formed by different cell types (A) Schematic diagram of invadopodia/podosomes (B) Invadopodia formation by MDA-MB-231 human invasive breast cancer cells. The cells were cultured on rhodamine-gelatin-coated coverslips and stained with phalloidin to detect invadopodia that are enriched with actin filaments (F-actin). Upper and lower panels are confocal images showing XY and XZ sections, respectively. Invadopodia were observed as dot-like structures containing F-actin, which degrade the rhodamine-gelatin matrix, resulting in the loss of gelatin fluorescence in the region of the invadopodia (arrowheads). (C) Podosomes formed by NIH3T3 cells transformed by constitutively active Src (NIH3T3 src). Parental NIH3T3 and NIH3T3 src cells were cultured and stained as described in (A). NIH3T3 src cells, but not parental NIH3T3 cells, form podosomes, which are observed as donut-like actin structures and colocalized with the gelatin degradation sites (arrowheads). (D) Podosome formation of macrophages and osteoclasts. RAW264.7 cells were cultured in the presence of lipopolysaccharide (LPS) (100 ng/ml) or RANKL (10 ng/ml) for 72 h to induce differentiation into the macrophages or osteoclasts, respectively. Cells were stained with phalloidin and 4',6-diamidino-2-phenylindole (DAPI). Macrophages form podosomes that often organize into large clusters associated with the gelatin degradation sites (arrowheads). Osteoclasts form a dense circumferential band of F-actin, called the sealing zone (yellow arrowheads), and clusters of podosomes that are observed inside the sealing zone (white arrowheads). A large gelatin degradation region was observed under these structures.

Metastatic dissemination of cancer cells is the leading cause of mortality in patients with malignant cancers [1, 2]. Cancer cells need to degrade the extracellular matrix (ECM), which exists in the basement membrane, tumor stroma, and blood vessel walls, to emigrate from original tumor sites and invade adjacent tissues, and to eventually form metastatic sites at distant organs [3]. These processes seem to be facilitated by the formation of invadopodia, which are ventral membrane protrusions with ECM degradation activity formed by invasive cancer cells [4-8] (Fig. 1A and B). The ability of cancer cells to form invadopodia is closely related to their invasive and metastatic properties [9-11]. Additionally, during intravasation, invadopodia-like protrusions in cancer cells were observed *in vivo* by intravital imaging [12]. Furthermore, a recent study showed that invadopodia perforate the native basement membrane, allowing the invasive cancer cells to invade into the stroma [13]. Oncogene-transformed fibroblasts and cells of monocyte lineage also form functionally similar structures called podosomes that have ECM degradation activity (Fig. 1C and D). The podosomes of macrophages/osteoclasts are used not only to elicit their physiological functions, but also to help cancer cells achieve efficient metastasis. Therefore, invadopodia/podosomes and their molecular regulators are considered as potential targets in the development of therapeutic strategies for cancer invasion and metastasis.

To date, many components of invadopodia have been reported, including proteins involved in the regulation of the actin cytoskeleton, cell signaling, cell-ECM adhesion, ECM degradation, and membrane remodeling [8, 14]. We and other researchers have previously proposed that invadopodia formation occurs in several steps [9, 13, 15, 16]. Invadopodia precursors are assembled by actin polymerization machinery in response to extracellular stimuli. These structures are then stabilized by additional actin polymerization, and finally they gather matrix metalloproteinases to mature into functional invadopodia, which contain microtubules and intermediate filaments in addition to actin filaments. How these events occur at restricted sites on the plasma membrane of invasive cancer cells, however, is obscure. Recently, several studies regarding the role of membrane lipids in the regulation of invadopodia/podosome formation have been reported.

## LIPID RAFTS AND CAVEOLIN-1 IN INVADOPODIA FORMATION

Lipid rafts are cholesterol-and sphingolipid-enriched membrane microdomains that are also referred to as lipid microdomains or detergent-resistant membranes (DRM). Lipid rafts have been implicated in a number of critical cellular processes, such as membrane transport and signal transduction [17, 18], as well as several pathological conditions, including cancer progression [19–21]. Caveolin-1 is a ubiquitously expressed scaffolding protein that is enriched in caveolae, which are subtypes of lipid rafts [22, 23]. Caveolin-1 is involved in several cellular functions such as endocytosis, vesicular transport, and signal transduction [23, 24].

Both we and Caldieri et al. recently reported that invadopodia are lipid raft-enriched domains in human breast cancer and melanoma cells [10, 25]. We also observed that lipid rafts were enriched at podosomes formed by Src-transformed fibroblasts (unpublished observations). The inhibition of lipid rafts by the depletion or sequestration of membrane cholesterol, or the blocking of glycosphingolipid synthesis, has been shown to impair invadopodia formation and function [10, 25]. Time-lapse observation revealed that lipid raft membranes are actively trafficked and internalized around invadopodia, which indicates the possible involvement of lipid rafts in the transport of invadopodia components [10]. Several invadopodia components involved in actin polymerization and membrane trafficking, including neural Wiskott-Aldrich syndrome protein (N-WASP), dynamin-2, and Arf6, are known to localize at lipid rafts [17, 26, 27]. Therefore, lipid rafts may act as platforms for localizing and activating these molecular machineries at the sites of invadopodia formation, which results in focalized ECM degradation.

The 2 studies also revealed that caveolin-1 is an essential regulator of the invadopodia-mediated degradation of ECM, which indicates that caveolin-1 plays an essential role in cancer cell invasion [10, 25]. Indeed, at least in breast cancer cell lines, caveolin-1 expression is predominantly observed in invasive cell lines and well correlated with invadopodia activity [10]. In melanoma cells, caveolin-1 functions at invadopodia through cholesterol transport to maintain proper levels of plasma membrane cholesterol [25]. Meanwhile, caveolin-1 is primarily involved in the transport of lipid raft-associated membrane type I matrix metalloproteinase (MT1-MMP), an invadopodia-enriched matrix metalloproteinase that is responsible for the ECM degradation activity of invadopodia [10]. Although further studies are needed to elucidate the precise functions of caveolin-1 in invadopodia formation, these findings imply that caveolin-1 plays multiple roles in the trafficking of invadopodia components. Clinical studies showed that the increased expression of caveolin-1 is correlated with the presence of metastasis and poor prognosis in several human cancers [28, 29]. Taken together, blocking the functions of lipid rafts and caveolin-1 should be an approach to targeting invadopodia-mediated cancer cell invasion.

## PHOSPHOINOSITIDE SIGNALING IN INVADOPODIA FORMATION

Phosphoinositides are membrane lipids that play multiple important roles in diverse cellular functions, such as membrane trafficking, signal transduction, cytoskeletal remodeling and deformation of the plasma membrane [30, 31]. Phosphoinositides are reversibly phosphorylated at the 3 different positions of the inositol headgroup. This generates 7 different species of phosphoinositide, namely, phosphatidylinositol 3-phosphate (PI3P), phosphatidylinositol 4-phosphate (PI4P), phosphatidylinositol 5-phosphate (PI5P), phosphatidylinositol 3,4-bisphosphate [PI(3,4)P2], phosphatidylinositol 4,5-bisphosphate [PI(4,5)P2], phosphatidylinositol 3,5-bisphosphate [PI(3,5)P2], and phosphatidylinositol 3,4,5-trisphosphate [PI(3,4,5)P2]. There are a large number of enzymes that phosphorylate, dephosphorylate, or hydrolyze phosphoinositides to locally and temporarily regulate the levels of these phosphoinositide species [32-34]. As a result, each phosphoinositide has unique cellular distributions, which allows them to selectively recruit or activate target proteins that have specific phosphoinositide-binding domains [35]. PI(4,5)P2 also acts as a precursor of second messengers: phospholipase C (PLC) hydrolyzes PI(4,5)P2 to generate diacylglycerol (DAG) and inositol 1,4,5-trisphosphate (IP3) and phosphoinositide 3-kinase (PI3-kinase) phosphorylates PI(4,5)P2 to generate PI(3,4,5)P3 [30, 36]. Proteins that sever or depolymerize actin filaments *in vitro*, which include gelsolin, villin, cofilin, and profilin, are inactivated by PI(4,5)P2 localized at the plasma membrane [37]. Conversely, proteins including vinculin, talin, ezrin/radixin/moesin (ERM) proteins, Wiskott-Aldrich syndrome protein (WASP)/N-WASP, and α-actinin, which link actin filaments to each other or to the plasma membrane, are activated by this lipid [37, 38].

We recently reported that PI(4,5)P_2_ is enriched at invadopodia and blockage of the PI(4,5)P_2_ function suppresses invadopodia formation and ECM degradation by invasive human breast cancer cells [39]. We also found that a kinase generating PI(4,5)P_2_, phosphatidylinositol-4-phosphate 5-kinase type Iα (PIPK Iα), accumulates at the invadopodia and that a knockdown of PIPK Iα inhibits invadopodia formation. Importantly, the knockdown of PIPK Iα only affects a pool of PI(4,5)P_2_, which is locally and newly produced by PIPK Iα. The knockdown of PIPK Iα resulted in only a slight decrease in the total amount of PI(4,5)P_2_, and did not affect the PI3-kinase signaling pathway, in which PI(4,5)P_2_ acts as a major substrate. Therefore, PI(4,5)P_2_ seems to exert its function via direct regulation of its own targets. Our previous study showed that N-WASP and its activators, including Nck, are critical regulators of actin polymerization at the invadopodia core structures [9]. Because the activation of N-WASP is regulated by the amount of PI(4,5)P_2_ on the plasma membrane [40], N-WASP is the most probable candidate for the PI(4,5)P_2_ target. A recent study identified the existence of the reciprocal interdependence between Nck and PI(4,5)P_2_ for regulation of N-WASP activity [41]. Nck is required for N-WASP-dependent actin polymerization induced by PI(4,5)P_2_ and Nck also stimulates PI(4,5)P_2_ production via recruitment of PIPK Iα. Considering that Nck and PI(4,5)P_2_ are essential for invadopodia formation [9, 39], these components may interdependently activate N-WASP to assemble invadopodia structures. Other invadopodia components, such as cofilin and dynamin-2, are also regulated by PI(4,5)P_2_ [31]. Arf6 is known to activate PIPK Iα for PI(4,5)P_2_ production [42]. Interestingly, synaptojanin-2, a PI(4,5)P_2_/PI(3,4,5)P_3_ phosphatase, is accumulated at invadopodia and required for invadopodia formation, which implies that this enzyme regulates the turnover of PI(4,5)P_2_ at invadopodia [43]. Taken together, local PI(4,5)P_2_ metabolism occurs at invadopodia and this seems to be a critical event to coordinate localization and activation of the invadopodia components. Classic biochemical studies have shown that phosphoinositide metabolism preferentially occurs within lipid raft fractions [44, 45]. Additionally, it has been shown that PI(4,5)P_2_-dependent actin polymerization induced by N-WASP and Arp2/3 complex is initiated at the surface of the lipid raft-enriched membranes [46, 47]. Therefore, lipid rafts and phosphoinositides may cooperatively regulate invadopodia formation.

**Figure 2:**
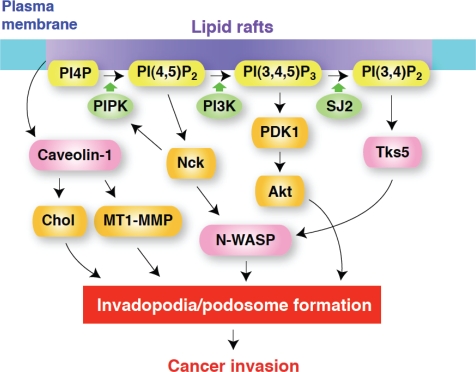
A model for the regulation of invadopodia/podosome formation by membrane lipids Lipid rafts may act as platforms for the recruitment of components of invadopodia/podosomes and for localized signaling by phosphoinositides. Caveolin-1 enriched in lipid rafts plays a role in cholesterol transport to maintain the levels of plasma membrane cholesterol (Chol) and also in MT1-MMP transport for the maturation of invadopodia. PI(4,5)P2 generated by PIP kinase type Iα (PIPK) acts as a signaling molecule to locally activate several invadopodia components, including N-WASP, and also serves as a substrate of PI3-kinase (PI3K) for generation of PI(3,4,5)P3, which in turn regulates invadopodia formation, most likely through PDK1 and Akt. Nck is required for N-WASP dependent actin polymerization induced by PI(4,5)P2 and also stimulates PIPK for local enrichment of PI(4,5)P2. PI(3,4)P2, produced from PI(3,4,5)P3 by the action of a specific phosphatase, possibly synaptojanin-2 (SJ-2), recruits Tks5/FISH to the plasma membrane, along with its binding partner N-WASP and other proteins involved in the formation of invadopodia/podosomes. It should be noted that the functions and requirements of these molecules may be slightly different between invadopodia and podosomes, as well as among cell types.

The PI3-kinases are a family of lipid kinases that phosphorylate phosphoinositides at the D-3 position of the inositol headgroup and thus produce D-3 phosphoinositides [48]. PI3-kinases mediate the signal transduction of extracellular stimuli and regulate diverse cellular events, such as mitogenesis, survival, membrane transport, and cell migration [36]. PI3-kinases are subdivided into 3 classes (I–III) in mammals on the basis of their enzyme domain structures and substrate specificity [33]. Uncontrolled activation of the PI3-kinase signaling pathway leads to several pathological phenomena, including tumorigenesis and tumor malignancies [36]. This is evidenced by the fact that the expression and activity of several members of the PI3-kinase signaling pathway are frequently altered in a variety of human cancers [49]. PI3-kinase activity is also required for invadopodia formation, as shown in invasive melanoma cells [50]. In line with this, we recently found that class IA PI3K catalytic subunit p110α is selectively involved in invadopodia formation in breast cancer cells, and that PDK1 and Akt mediate the signaling (manuscript in submission). The PIK3CA gene, which encodes p110α, is one of the most frequently amplified and mutated genes identified in human cancers [49, 51]. Several clinical studies revealed that mutations leading to the activation of the PIK3CA gene are associated with invasive and metastatic phenotypes, as well as poor prognosis [52-54]. Moreover, introduction of the mutant PIK3CA gene was reported to enhance the migration, invasion, and metastasis of breast cancer cells [55]. Therefore, p110α is considered as a promising molecular target for the intervention of malignant cancers, and it has led to the development of several specific inhibitors [56].

## CELLS OF MONOCYTE ORIGIN GENERATE PODOSOMES, WHICH CONTRIBUTE TO CANCER CELL INVASION AND OSTEOLYTIC BONE METASTASIS

Podosomes are F-actin-rich, dynamic adhesion structures found in Src-transformed cells (Fig. 1C) and the physiological context of monocyte-derived cells such as macrophages and osteoclasts (Fig. 1D). In the past decade, the generation of podosomes has been proven to be associated with the gene responsible for an X chromosome-linked immunodeficiency disease, Wiskott-Aldrich syndrome (WAS). As macrophages from patients with WAS have defects in generating podosomes and polarization of the cell [57], these structures are thought to be important for chemotactic migration and/or the invasion of macrophages. Actually, the product of the gene (i.e., WASP) and its ectopic analogue, N-WASP, were shown to be indispensable for actin polymerization at podosomes via activating the Arp2/3 complex [57–61].

It is well established that the neoplastic properties of cancer cells are affected by interactions with the tumor microenvironment [62]. Tumor-associated macrophages (TAMs) have been implicated in tumor progression, metastasis, and poor prognosis in several human cancers [63, 64]. A paracrine loop between macrophages and cancer cells has been proven to facilitate cancer cell migration and invasion both *in vitro* and *in vivo*, confirming the vicious role of TAMs [65, 66]. Cancer cells stimulate the invasion of macrophages by secreting colony stimulating factor-1 (CSF-1), which in turn causes the macrophages to stimulate invasion of the cancer cells by secreting epidermal growth factor (EGF). EGF and CSF-1 are shown to stimulate invadopodia formation in cancer cells and podosome formation in macrophages, respectively [6, 9]. Therefore, the paracrine loop between cancer cells and TAMs may promote cancer progression [63, 64] partly via the formation of invadopodia/podosomes.

Osteoclasts are highly specialized multi-nucleated cells that are differentiated from the monocyte/macrophage precursors on the bone surface in response to CSF-1 and receptor activator of nuclear-factor-κB ligand (RANKL). During the differentiation into mature cells, osteoclasts reorganize the actin cytoskeleton to form a dense circumferential band of F-actin (Fig. 1D). This ring forms a tight adhesive contact (the sealing zone) that defines a subcellular environment (which is known as a resorption pit or lacuna) into which H+ and lytic enzymes are secreted, thereby allowing effective erosion of the bone [58, 67]. The fully mature osteoclast can detach from the bone and move away from the resorption lacuna to participate in several rounds of resorption, which require podosome-associated cell motility [67, 68]. One of the upstream regulators of WASP, the cytoplasmic kinase Src is essential for osteoclast activity *in vivo*, because Src knockout mice suffer from severe osteopetrosis caused by deficient osteoclast activity [69]. Osteoclasts derived from such mice cannot adhere and spread properly, and fail to give rise to mature sealing zones when attached to the bone. Bone metastases from breast cancer are typically osteolytic and cause destruction of the bone [70]. Breast cancer cells augment the activity of bone resorption via promoting the differentiation and podosome formation of osteoclasts by secreting transforming growth factor-beta (TGF-β), tumor necrosis factor-alpha (TNF-α), interleukins (ILs), and parathyroid hormone-related protein (PTHrP), which leads to osteolytic bone metastasis.

## PRODUCTS OF PI3-KINASE REGULATE PODOSOME FORMATION

PI(3,4,5)P_3_, which is present in negligible amounts under resting conditions, is produced in the plasma membrane in response to extracellular stimuli; it is synthesized from PI(4,5)P_2_ by the action of class I PI3-kinase [36]. PI(3,4)P_2_ is produced by the action of class I and II PI3-kinase on PI(4)P or via the dephosphorylation of PI(3,4,5)P_3_ by PI(3,4,5)P_3_ 5-phosphatases such as SHIP2 and synaptojanin-2 [31]. These locally produced PI(3,4,5)P_3_ and PI(3,4)P_2_ recruit cytosolic proteins to the plasma membrane [35]. For example, the adhesion-mediated production of PI(3,4,5)P_3_ stimulates protein complex formation, including PI3-kinase, Src, and gelsolin, which is mediated by direct interactions between PI(3,4,5)P_3_ and the Src-homology 2 (SH2) domains of PI3-kinase and/or Src [71]. Furthermore, phosphoinositides are able to modulate the functions of small GTPases of the Arf and Rho families, such as Rho, Rac, and Cdc42; these proteins are shown to be involved in podosome formation [72-74]. This modulation occurs via the action of guanine nucleotide exchange factors (GEFs) or GTPase-activating proteins (GAPs) such as Tiam1, Vav1, and ASAP1 on these proteins; all these factors possess pleckstrin homology (PH) domains via which they interact with PI(4,5)P_2_ and/or PI(3,4,5)P_3_ [75, 76].

We investigated the localization of different species of phosphoinositides using various phosphoinositide-binding PH domains [77]. We demonstrated that PI(3,4)P2 is highly enriched in podosomes compared to the relatively diffused localization of PI(3,4,5)P3, which is also found in lamellipodia and intracellular vesicles. What is intriguing is that excessive expression of the PH domain of Tapp1, which binds to PI(3,4)P2, as well as the PH domain of Akt, which binds both to PI(3,4)P2 and PI(3,4,5)P3, significantly suppressed podosome formation. This effect is thought to occur through sequestering those lipids by the domains, because the amount of protein expressed in a cell tends to correlate with the suppression effect. Furthermore, we found that PI(3,4)P2 is synthesized by PI3-kinase and synaptojanin-2 in the vicinity of the focal adhesions, and that this phosphoinositide triggers the recruitment of a protein complex that includes Tks5, Grb2, and N-WASP, which results in the conversion of the adhesion sites to podosomes [77, 78]. Our results support the essential role of synaptojanin-2 in glioma cell migration and invasion [43], although the localization of PI(3,4)P2 in glioma cells has not been determined. Tks5 is an adaptor protein with an N-terminal phox homology (PX) domain, which was originally identified as an Src substrate [79]. Both Tks5 and its relative, Tks4, have been shown to play important roles in podosome formation, matrix degradation, and tumor growth *in vivo* [80–83]. Recently, they have been shown to mediate the generation of reactive oxygen species (ROS) at the invadopodia of cancer cells, which is required for invadopodia formation and cancer cell invasion [84, 85]. Moreover, Tks5 binds to supervillin, a lipid raft-enriched protein which is involved in integrin recycling, cell motility, and invadopodia formation [86-88], which suggests that they play roles as versatile regulators of invadopodia/podosomes. As the PX domain of Tks5 binds to PI(3,4)P2, and this interaction is essential for podosome formation downstream of Src [77, 82, 83], targeting this interaction would be a promising therapeutic strategy for the selective intervention of cancer cell invasion and metastasis.

## CONCLUDING REMARKS

As described above, accumulating evidence leaves us in no doubt that invadopodia/podosomes play a pivotal role in the invasion and metastasis of cancer cells. Moreover, podosomes formed by TAMs and osteoclasts in the tumor microenvironment seem to play supportive roles for cancer invasion and metastasis. The organization and components of the plasma membrane, such as lipid rafts and phosphoinositides, regulate the formation of invadopodia/podosomes. Therefore, targeting the molecular components of these structures, which include membrane lipids and their synthetic pathways, will contribute to the development of new strategies for the treatment of cancer invasion and metastasis.

One question that still remains answered is how lipid raft formation/degradation and phosphoinositide turnover are spatiotemporally regulated at invadopodia/podosomes. It is evident that invadopodia/podosomes are formed through several functional steps. Therefore, lipid rafts and phosphoinositide species may have distinct functions at different stages of invadopodia/podosome formation. Furthermore, although invadopodia and podosomes seem to share basic molecular components and functions, i.e., ECM degradation, their morphologies are quite different, even among cell types. If membrane lipids determine the site of invadopodia/podosome assembly, they may be critical determinants for the morphology, and most likely the function, of these structures. Further studies will be needed to address these questions.
